# A Comparative Study of Selected Physical and Biochemical Traits of Wild-Type and Transgenic Sorghum to Reveal Differences Relevant to Grain Quality

**DOI:** 10.3389/fpls.2017.00952

**Published:** 2017-06-07

**Authors:** Roya J. Ndimba, Johanita Kruger, Luke Mehlo, Alban Barnabas, Jens Kossmann, Bongani K. Ndimba

**Affiliations:** ^1^iThemba LABS, National Research FoundationCape Town, South Africa; ^2^Institute for Plant Biotechnology, University of StellenboschMatieland, South Africa; ^3^Department of Food Science and Institute for Food Nutrition and Well-Being, University of PretoriaPretoria, South Africa; ^4^Enterprise Creation for Development Unit, Council for Scientific and Industrial ResearchPretoria, South Africa; ^5^Agricultural Research Council, Infruitec-NietvoorbijStellenbosch, South Africa; ^6^Proteomics Unit, Department of Biotechnology, University of the Western CapeBellville, South Africa

**Keywords:** sorghum, transgenic, amino acid profile, protein body, grain quality traits

## Abstract

Transgenic sorghum featuring RNAi suppression of certain kafirins was developed recently, to address the problem of poor protein digestibility in the grain. However, it was not firmly established if other important quality parameters were adversely affected by this genetic intervention. In the present study several quality parameters were investigated by surveying several important physical and biochemical grain traits. Important differences in grain weight, density and endosperm texture were found that serve to differentiate the transgenic grains from their wild-type counterpart. In addition, ultrastructural analysis of the protein bodies revealed a changed morphology that is indicative of the effect of suppressed kafirins. Importantly, lysine was found to be significantly increased in one of the transgenic lines in comparison to wild-type; while no significant changes in anti-nutritional factors could be detected. The results have been insightful for demonstrating some of the corollary changes in transgenic sorghum grain, that emerge from imposed kafirin suppression.

## Introduction

Nearly 800 million of the world's population suffer from undernourishment, with the highest levels (~23%) found in Sub-Saharan Africa (FAO, IFAD, and WFP, 2015). Here, high rates of population growth coupled with severe climatic conditions often result in a chronic supply-demand mismatch (Knox et al., 2012). Grain sorghum [*Sorghum bicolor* (L) Moench], is one of Africa's most prized indigenous cereals that has been relied upon for centuries as a major food security crop (Dicko et al., 2006). Approximately 43% of all major staple foods produced on the continent are known to incorporate some aspect of sorghum grain (Etuk et al., 2012), and as a result, the crop is frequently cited as Africa's second most important cereal, after maize (Hassan, 2015). However, an over-reliance on sorghum as a basic staple is not recommended due to several nutritional shortcomings associated with its grain. Firstly, sorghum's dominant storage proteins, the kafirins, are difficult to digest and are moreover deficient in the essential amino acids lysine, methionine and tryptophan (Shewry, 2007; Wong et al., 2009). Secondly, some sorghum grain types contain high levels of anti-nutrients such as tannins and phytate (Reddy et al., 1982; Gilani et al., 2012), which are known to strongly bind proteins and essential minerals, leading to reduced bioavailability, and hence compromised levels of nutrient uptake.

Given that poor communities subsisting on monotonous cereal-based diets, like sorghum, are vulnerable to malnutrition, significant research has been directed toward improving the nutritional value of sorghum grain (Zhao, 2008; Reddy et al., 2010). Grootboom et al. (2014), used RNA interference (RNAi) technology to suppress the expression of select kafirin subclasses, which resulted in transgenic sorghum grain with improved protein digestibility levels of up to 53%. Although, this approach has been recognized as an effective strategy for increasing the protein digestibility (and hence the nutritional value) of sorghum grain, these “improved” transgenic lines have not yet been commercialized or cultivated. This is likely a consequence of the strict legislative requirements involved in the pre-approval of genetically modified (GM) food crops before general or commercial release (Shepherd et al., 2006; García-Villalba et al., 2008). Undoubtedly, a key concern is to ensure that the transgenic food item is as safe and nutritious as its non-transgenic counterpart, and poses no potential new risks to the consumer or the environment (Chassy, 2010; Jiang and Xiao, 2010).

Several studies have been conducted which compare transgenic and conventionally bred lines of major food crops such as maize (Ridley et al., 2002; George et al., 2004; Levandi et al., 2008; Barros et al., 2010), wheat (Baker et al., 2006; Baudo et al., 2006), rice (Li et al., 2007; Jiang and Xiao, 2010; Gayen et al., 2013), potatoes (Shepherd et al., 2006; El-Khishin et al., 2009), and soybean (García-Villalba et al., 2008; Zhu et al., 2008). Thus, far, however, no such comparable studies have been completed on transgenic sorghum. In the present work an attempt will be made to investigate aspects of substantial equivalence between transgenic sorghum grain and its non-transgenic parental counterpart. The approach will involve a comparative analysis of key physical and biochemical characteristics of the grains that have an important impact on grain quality and/or nutritional value. The key physical grain quality traits that will be analyzed include a comparative evaluation of grain weight, grain hardness, and protein body morphology. For assessing differences in the biochemical composition, a comparative study of the amino acid composition and key anti-nutrients (such as condensed tannins and phytate) will be carried out. The results of this study will be important for highlighting some of the key differences that distinguish the transgenic grain from the wild-type and further, whether or not these differences have significant biological or nutritional consequences.

## Materials and methods

### Plant materials

Two independent transgenic sorghum lines, featuring the pABS042 construct, for the targeted suppression of γ-kafirin-1 (27 kDa) and γ-kafirin-2 (50 kDa) were assessed in the present study. Previous analyses found complete suppression of the targeted kafirin proteins and a significant improvement (of up to 37%) in the *in vitro* protein digestibility of the transgenic grains (Grootboom et al., 2014). The transgenic lines represent the fifth generation of self-crossing progenies from T_0_ sorghum transformants, which were originally produced using particle bombardment as described in Grootboom et al. (2014). Plants from each independent transgenic line (hereafter referred to as 42-1 and 42-2) and the parental control cultivar P898012 (hereafter referred to as WT), were grown in pots in a containment glasshouse located at the Centre for Scientific and Industrial Research (CSIR) Biosciences Division (Pretoria, South Africa). Environmental conditions were controlled (12-h photoperiod, 25°C/20°C day/night, ~50% humidity, 800 μE/s/m^2^ irradiance), with a minimum of three biological replicates per line. At each occasion of watering, the plant arrangement in the glasshouse was randomized to counter any possible positional effects due to the microhabitat, and at anthesis, the panicle of each plant was bagged to prevent outcrossing. At full maturity, the grains were hand-harvested from each plant, and manually cleaned to remove all glumes, damaged grains, and other extraneous matter. For the physical characterization of the grain, whole kernels, separately derived from individual plants of each genotype were used to make up the sample. However, for the chemical analyses, the whole kernels had to be ground into fine flour. To reduce biological variation and to maximize the limited quantity of sample material available, equal portions of grain from individual plants from each genotype were pooled and homogenized to fine flour, using a standard laboratory mill. Milled grain flour was used immediately for the intended analysis or stored at −20°C in airtight containers until analyzed.

### Physical characteristics

#### Kernel weight

For each line, the mass of 100 randomly selected kernels was determined using a 10^−4^ precision balance. This measurement was repeated three times for three biological replicates per line, to obtain the mean 100-kernel weight ± *SD*.

#### Grain hardness

To evaluate differences in grain hardness, the floater test (Hallgren and Murthy, 1983) was used, where 100 randomly selected kernels from each line (replicated 3 times) were placed in a solution of sodium nitrate with a specific gravity of 1.250 g/mL (as measured with a hydrometer) and the percentage of floating kernels (low density) was determined. As a complement to this test, a visual inspection of the grain endosperm texture was also carried out. Individual kernels (20 randomly selected from each line, replicated 3 times) were longitudinally sectioned through the middle and subjectively categorized as a floury (completely opaque) or an intermediate (partly corneous/partly opaque) phenotype using ICC Standard No.176 (ICC, 2008). The percentage of floury endosperm phenotypes found for each line was then tabulated and compared.

#### Protein body morphology

Transmission electron microscopy (TEM) was used to investigate the morphology of the grain protein bodies. In brief, segments of the peripheral endosperm (~1–2 mm^3^ pieces) were sectioned from randomly selected mature grains of each line, and fixed in a 0.1 M sodium cacodylate buffer containing 3% (v/v) glutaraldehyde (pH 7.2) for 24 h at room temperature. The samples were then postfixed in 2% (v/v) osmium tetraoxide at 4°C for 24 h, dehydrated in a graded ethanol series and embedded in Agar Low Viscosity Resin for 16 h at 70°C. Ultrathin (60–90 nm) sections of resin embedded samples were cut using an ultramicrotome (Leica EM UC7) fitted with a Diatome diamond knife and double stained with 2% (w/v) uranyl acetate (8 min) and 0.1% (w/v) lead citrate (5 min). Protein bodies located in the sub-aleurone layer of the grain endosperm tissue were imaged using a FEI Tecnai™ G2 transmission electron microscope, operating at an accelerating voltage of 200 kV (Electron Microscope Unit, University of Cape Town).

### Biochemical characteristics

#### Total amino acid composition

The protein-bound amino acid content was measured according to the Pico-Tag reverse-phase high performance liquid chromatography (RP-HPLC) procedure (Bidlingmeyer et al., 1984) at the South African Grain Laboratory (SAGL) (Pretoria, South Africa). In brief, sorghum samples were hydrolysed in 6N HCl for 24 h, after performic acid oxidation (for the analysis of cysteine and methionine) or without previous oxidation (for all other amino acids). The liberated amino acids were then derivatized before separation and quantification by means of RP-HPLC using a Waters™ Pico-Tag C18 column (3.9 × 150 mm) and the relevant amino acid standards.

#### Anti-nutrient levels

The condensed tannins and phytate content were measured in duplicate for two independent samples of each sorghum genotype. To determine the condensed tannin contents, a modified vanillin-HCl method was followed (Price et al., 1978), where the tannins were extracted with acidified methanol. For each sample, the absorbance of sample blanks were subtracted to compensate for non-tannin pigments related to grain color. The final concentration of condensed tannins in each sample was determined against a gallic catechin standard, and expressed in mg catechin equivalents (CE) per g of wholegrain sorghum flour. The phytate levels were determined using an indirect quantitative assay based on anion-exchange chromatography (Frubeck et al., 1995) following extraction with 2.4% conc. HCl. The final phytate content was expressed in terms mg/g wholegrain sorghum flour.

### Statistical analysis

Unless otherwise stated, investigated parameters were analyzed in triplicate and the results expressed as the mean ± *SD*. Data were subjected to analyses of variance (ANOVA) and means compared by the Fisher Least Significant Difference (LSD) test (*p* < 0.05) using *Statistica* for Windows Version 13.0 (StatSoft Inc., USA).

## Results and discussion

### Differences in the physical traits of the transgenic vs. WT grains

**Figure 1:**
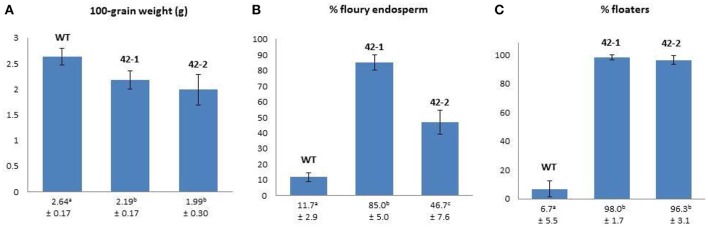
A comparison of 100-grain weight **(A)**, % floury endosperm **(B)**, and % floaters **(C)** between wild-type (WT) and transgenic sorghum genotypes 42-1 and 42-2. Values below the bars on the charts correspond to the mean ± *SD*. Means sharing the same superscript are not significantly different at *p* < 0.05.

The results pertaining to differences in the physical traits of grain from the two transgenic lines (42-1, 42-2) and their non-transgenic parental counterpart (WT) are presented in Figure 1. For the mean 100-grain weight, both transgenic lines were observed to have a lower weight in comparison to WT, but a statistically significant reduction could only be ascribed to the grains in line 42-2. In the case of the proportion of floury endosperm types and the percentage of floating kernels, both transgenic lines were found to have significantly increased levels of these characteristics in comparison to WT.

According to the literature, physical traits such as grain weight, density, and endosperm texture are important indicators of quality for sorghum because of their impact on grain hardness. Hard grains tend to be heavy (>2.0 g 100-grain weight) with a low percentage of floaters (<40%), and a corneous to intermediate endosperm texture (Gomez et al., 1997). For large scale production and processing, hard sorghum grains are generally preferred because of their association with reduced post-harvest losses (due to breakage and/or spoilage) and enhanced milling yields (Jambunathan et al., 1992; Simonyan et al., 2007).

The high percentage of floating grains amongst the transgenic lines (>96%) indicated a significant decrease in the grain density in comparison to the WT. The rationale informing the use of the sodium nitrate solution with a specific gravity of 1.25 g/mL is that this value is approximately equal to the average density of a wide range of sorghum kernels (Kirleis and Crosby, 1981). Thus, in this solution, kernels of lower density than average will float, while those of greater density will sink (Gomez et al., 1997). It is therefore clear that the transgenic grains are not only less dense than the WT, but are also less dense than the average known for sorghum grain. The reason for this decreased density is likely attributable to the significant increase in the percentage of floury endosperm types. The floury endosperm phenotype is associated with loosely packed starch granules that accommodate more air spaces (Rooney and Miller, 1982; Hoseney, 1994). It is therefore likely that the reduced density in the transgenic grain is attributable to the inclusion of more air spaces within the grain endosperm, which results in the characteristic opaque or floury visual appearance of this endosperm structure.

The increased percentage of floury endosperm phenotypes in the transgenic lines was commensurate with similar findings reported previously (Da Silva et al., 2011; Grootboom et al., 2014). Amongst the transgenic lines, a significant difference in the average number of floury endosperm types, was observed, with the highest percentage found in line 42-1 (85%), which was close to twice the value of that found in line 42-2 (47%). The floury endosperm phenotype is generally associated with improved nutritional qualities, such as improved protein digestibility and essential amino acid content (Singh and Axtell, 1973; Tesso et al., 2006; Da Silva et al., 2011). However, these nutritional gains are often overshadowed by the increased susceptibility of these softer floury grains to post-maturity losses and damage due to molding and insect infestation (Waniska and Rooney, 2002).

**Figure 2:**
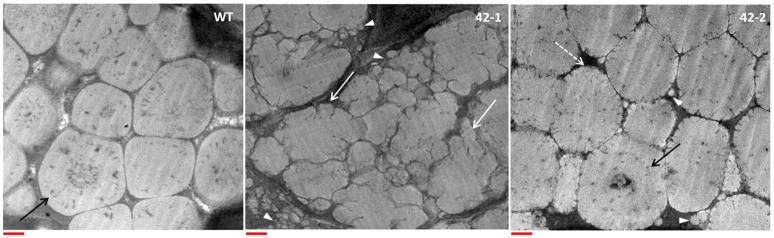
Representative TEM images of protein bodies in the subaleurone layer of the wild-type (WT) and transgenic sorghum lines (42-1, 42-2). Black arrow points to internal concentric ring structure present in WT and some transgenic protein bodies. White arrows point to irregularly shaped protein bodies with invaginations at the periphery. “Cracked” peripheral structure of transgenic protein bodies, pointed out with white dotted arrow. White arrowheads point out the presence of small protein bodies (<0.5 μm diameter) in the transgenic lines. Scale bar represents 0.5 μm.

Morphological features of the grain protein bodies were studied using TEM analysis, the results of which are shown in Figure 2. In the earlier work of Grootboom et al. (2014), the protein bodies of the transgenic lines transformed with the ABS042 construct did not deviate much in terms of overall shape and size, as compared to the protein bodies found in the grain of the non-transgenic parent, P898012. This finding is consistent with similar studies featuring transgenic sorghum with targeted γ-kafirin suppression, such as the ABS 149 line (Da Silva et al., 2011) and the TX430 line (Kumar et al., 2012). In the present work, however, some important deviations in the morphology of the protein bodies in the transgenic lines were detected. Firstly in terms of size, no protein bodies of less than 0.5 μm diameter were found in the WT. In contrast, both transgenic lines were found to have protein bodies (of varying number) that were diminutive in size (<0.5 μm) in comparison to the WT. The reduced size of the protein bodies translate to a reduced surface to volume ratio, which would favor enhanced proteolytic digestion. Secondly in terms of shape, the most extreme change of morphology was evident in transgenic line 42-1. WT protein bodies conformed to the expected highly-ordered polygonal structure, with internal concentric rings, that is typical of normal sorghum. In contrast the protein bodies of line 42-1, were highly irregular in shape, with no internal concentric ring structures, and with deep invaginations present at the periphery. In line 42-2, the protein bodies were closer in morphology to the WT, exhibiting a regular overall polygonal shape, with some distinctive patterns of internalized concentric rings. The only features that served to differentiate the protein bodies of transgenic lines 42-2 from WT, were the small peripheral indentations that gave the boundary region a cracked appearance. The highly misshapen protein body morphology of line 42-1 is commensurate with the structural changes found in protein bodies of high-lysine Ethiopian landraces (Singh and Axtell, 1973; Oria et al., 2000) and transgenic lines featuring both γ- and α-kafirin suppression (Da Silva et al., 2011; Grootboom et al., 2014). It may therefore be speculated that the intended γ-kafirin suppression in line 42-1 manifests itself in a unique way that is aligned to the genetic programming in the floury endosperm phenotypes, but is distinctly different from other “related” transgenic lines with targeted γ-kafirin suppression only. This result warrants further investigation, particularly into the storage protein expression profile of 42-1 grain, which may provide vital clues into the determining factors that may underlie this “novel” phenotype.

### Comparative differences in the biochemical traits of the transgenic vs. WT grains

#### Amino acids analysis

Data depicting the protein-bound amino acid content of the WT and two transgenic sorghum lines is presented in Table 1. A number of important variations that serve to distinguish the transgenic lines from the WT were observed. Firstly, in comparison to WT, transgenic line 42-1 featured more significant changes in amino acid composition compared to line 42-2. In line 42-1 the concentration of 9 out of 17 amino acids were determined to be significantly different from the WT, whereas for line 42-2 this was the case for only 4 amino acids. Three amino acids that were determined to be significantly reduced in both transgenic lines in comparison to WT were tyrosine, isoleucine, and phenylalnine. The sorghum kafirins are known to contain relatively higher amounts of tyrosine, isoleucine and phenylalanine in comparison to other grain storage proteins (Virupaksha and Sastry, 1968). It may therefore be suggested that the reduction in these three amino acids is reflective of the intended targeted suppression of kafirins in the transgenic grains by RNAi. Interestingly, in the case of arginine, a significant increase (25.3%) in comparison to WT was found for line 42-1; whereas, a significant decrease (11.8%) in comparison to WT was found for line 42-2. This important difference may be indicative of a shift in the relative amounts of non-kafirins that may be present in the grains, as non-kafirins tend to have high amounts of arginine (Lasztity, 1996). An increase in the relative amount of non-kafirin grain protein in line 42-1 seems particularly evident due to the significantly increased level of lysine (50.8%) in comparison to WT. Given that kafirins are virtually lysine-free (Belton et al., 2006), the improved lysine content of line 42-1, is likely due to an increased abundance of the more lysine-rich grain proteins, such as the albumins, globulins, and glutelins (Da Silva et al., 2011). The amino acid results have confirmed the distinctiveness of line 42-1 in comparison to its sister transgenic line (42-2), as well as to WT. Of key importance from a nutritional point of view, is the significant increase of lysine in line 42-1 grain. An increase in lysine serves as one of the most important indicators of improved nutritional value since this amino acid is most limiting in sorghum and most other cereals. Given that the observed increase in lysine for line 42-1 was not found before in the original study of Grootboom et al. (2014), it would be of interest to study this line more extensively to determine the long-term stability and precise molecular mechanisms underpinning this desirable trait.

**Table 1:** A comparison of the protein-bound amino acid content of the wild-type (WT) and transgenic sorghum genotypes 42-1 and 42-2, presented on a dry weight basis in mMol/100 g wholegrain flour.

**Amino acids**	**WT**	**42-1**	**42-2**	***p*-value**
MET^*^	2.10^a^ ± 0.16	2.06^a^ ± 0.23	1.99^a^ ± 0.24	0.804
CYS^*^	3.16^a^ ± 0.70	2.39^a^ ± 0.38	2.99^a^ ± 0.58	0.290
ASX	9.29^a^ ± 0.20	10.14^a^ ± 0.08	9.46^a^ ± 0.70	0.102
GLX	25.53^a^ ± 0.74	23.22^b^ ± 0.43	25.69^a^ ± 0.60	0.004
SER	7.94^a^ ± 0.51	7.15^a^ ± 0.13	7.71^a^ ± 0.23	0.062
GLY	7.63^a^ ± 1.00	7.77^a^ ± 0.36	7.52^a^ ± 0.57	0.903
HIS^**^	3.25^a^ ± 0.09	2.76^b^ ± 0.17	3.22^a^ ± 0.05	0.003
ARG^**^	5.09^a^ ± 0.35	6.38^b^ ± 0.27	4.49^c^ ± 0.02	0.0003
THR^*^	5.22^a^ ± 0.36	5.24^a^ ± 0.18	5.26^a^ ± 0.12	0.974
ALA	16.13^a^ ± 0.68	14.77^b^ ± 0.36	16.21^a^ ± 0.19	0.014
PRO	12.39^a^ ± 0.36	10.57^a^ ± 1.67	13.07^a^ ± 1.14	0.094
TYR^*^	3.49^a^ ± 0.17	2.60^b^ ± 0.27	2.92^b^ ± 0.29	0.013
VAL^*^	8.25^a^ ± 0.41	8.24^a^ ± 0.23	8.30^a^ ± 0.10	0.962
ILE^*^	5.64^a^ ± 0.29	5.06^b^ ± 0.20	5.21^b^ ± 0.11	0.038
LEU^*^	16.42*^a^* ± 1.13	14.27^b^ ± 0.44	16.06^a^ ± 0.32	0.023
PHE^*^	5.58^a^ ± 0.15	4.65^b^ ± 0.18	5.30^c^ ± 0.06	0.005
LYS^*^	2.38^a^ ± 0.24	3.59^b^ ± 0.07	2.34^*a*^ ± 0.15	0.000

Values are means (±SD) of three independent determinations. Means in rows sharing the same superscript are not significantly different at p < 0.05; Essential amino acids marked with asterisk (^*^). Conditionally essential amino acids are marked with double asterisk (^**^).

#### Anti-nutritional factors

A statistical comparison of tannin and phytate content revealed no significant differences between the transgenic samples and WT (Table 2). The genetic intervention therefore had no substantial impact on the levels of these anti-nutritional factors in the grain.

**Table 2:** A comparison of selected anti-nutritional factors present in wild-type (WT) and transgenic sorghum genotypes 42-1 and 42-2 wholegrain flour, on a dry weight basis.

**Anti-nutritional factor**	**WT**	**42-1**	**42-2**
Tannin (mg CE/g)	3.96 ± 0.42^a^	4.31 ± 0.72^a^	3.54 ± 0.47^a^
Phytate (mg/g)	8.74 ± 0.33^a^	8.43 ± 0.43^a^	8.34 ± 0.73^a^

*Values are means (±SD) of duplicate samples measured twice. Means in rows sharing the same superscript are not significantly different at p < 0.05*.

## Conclusion

This study has revealed a number of important differences in quality characteristics amongst and between two independent transgenic sorghum lines (featuring gamma kafirin suppression) and their wild-type counterpart. Importantly, transgenic grains were found to have a higher proportion of floury endosperm types, were conspicuously less dense and featured smaller protein body structures in comparison to wild-type grains. In terms of amino acid content, a significant increase in lysine was only found in transgenic line 42-1, which coincidentally also exhibited an extreme change in protein body morphology in comparison to that of WT. Many of the significant differences highlighted in this study were found to be consistent with observations made by other investigators interested in the high protein digestibility phenotype in sorghum varieties. A concern therefore remains that the softer endosperm phenotype may mean that the transgenic grains are more susceptible to abiotic and biotic stress in the field. Further studies are therefore needed to evaluate the field performance of these modified lines in conjunction with more extensive analysis of key grain quality traits, such as the significant increase in lysine in line 42-1 over a multi-year multi-location large-scale trial.

## Author contributions

RN: Carried out the bulk of the experimental work, compiled the results, drafted the manuscript. JoK: Responsible for the anti-nutrient experiments, gave scientific input on the structure and form of the manuscript, edited as necessary. LM: Provided access to the GM material, grew the plants at the containment facility, collected the samples, prepared material for the experiments, gave scientific input into the structure and form of the manuscript. AB: Instrumental in the preparation of samples for TEM analysis. JeK: provided lab infrastructure and funding in support of the experimental work performed at Stellenbosch. BN: provided lab infrastructure and funding in support of the experimental worked performed, in particular the amino acid analyses costs, and extensive editing and scientific input into the preparation of the final manuscript.

### Conflict of interest statement

The authors declare that the research was conducted in the absence of any commercial or financial relationships that could be construed as a potential conflict of interest.
